# Attitudes towards Advance Care Planning and Healthcare Autonomy among Community-Dwelling Older Adults in Beijing, China

**DOI:** 10.1155/2015/453932

**Published:** 2015-12-24

**Authors:** Ning Zhang, Xiao-hong Ning, Ming-lei Zhu, Xiao-hong Liu, Jing-bing Li, Qian Liu

**Affiliations:** ^1^Department of Geriatrics, Peking Union Medical College Hospital, Chinese Academy of Medical Sciences, Beijing 100730, China; ^2^Department of Neurology, Hebei Geriatric Hospital, Shijiazhuang, Hebei, China; ^3^Department of Geriatrics, Beijing Tongren Hospital, Capital Medical University, Beijing, China

## Abstract

*Objectives*. To investigate the preferences of ACP and healthcare autonomy in community-dwelling older Chinese adults.* Methods*. A community-based cross-sectional study was conducted with older adults living in the residential estate of Chaoyang District, Beijing.* Results*. 900 residents were enrolled. 80.9% of them wanted to hear the truth regarding their own condition from the physician; 52.4% preferred to make their own healthcare decisions. Only 8.9% of them preferred to endure life-prolonging interventions when faced with irreversible conditions. 78.3% of the respondents had not heard of an ACP; only 39.4% preferred to document in an ACP. Respondents with higher education had significantly higher proportion of having heard of an ACP, as well as preferring to document in an ACP, compared to those with lower education. Those aged <70 years had higher proportion of having heard of an ACP, as well as refusing life-prolonging interventions when faced with irreversible conditions, compared to those aged ≥70 years.* Conclusions*. Although the majority of community-dwelling older Chinese adults appeared to have healthcare autonomy and refuse life-prolonging interventions in terms of end-of-life care, a low level of “Planning ahead” awareness and preference was apparent. Age and education level may be the influential factors.

## 1. Introduction

The provision of optimum care for the aging population is dependent on the understanding of their views and values on healthcare issues, especially end-of-life issues. Advance care planning (ACP) is a process of reflection, discussion, and communication that enables a person to plan for their future medical treatment and other care, for a time when they are not competent to make or communicate decisions for themselves [1]. ACP generally takes one of two forms: (1) the advance directive or “living will,” a mechanism that allows individuals to catalog their preferences for future healthcare; (2) the durable power of attorney for healthcare or “healthcare proxy,” a document that assigns a surrogate to make medical decisions on behalf of a patient in the event of decisional incapacitation [2]. It has gained prominence internationally for perceived benefits in enhancing patient autonomy and ensuring that patients receive appropriate, high-quality end-of-life care, as well as reducing stress, anxiety, and depression in surviving family members [3]. In the United States, the Patient Self-Determination Act of 1991 aims to encourage patients to take the initiative to ensure that their values are respected at the end of their life [4]. This legislation is letting US society think over urgency of the way to deal with end-of-life care and decision-making [5]. Nowadays, up to 70% of community-dwelling older adults in the United States have completed an advance directive [6], and data from the Health and Retirement Study showed that the elderly Americans who had prepared advance directives received care that was strongly associated with their preferences [7].

China is a country with the largest population in the world and is facing a growing aging population, increased incidences of cancer, and a huge number of terminally ill patients [8]. Therefore, it is imperative to develop palliative care and implement ACP in the country. In recent years, there has been growing evidence from Hong Kong suggesting that Chinese older people residing in residential care homes are well aware of the anticipated death and welcome the opportunity to discuss issues related to end-of-life care and death preparation [9]. However, despite substantial international literature on ACP [10], there is a paucity of information on the attitudes of the elderly citizens towards ACP in mainland China. It is with this view that we conducted this study with the following objectives: (1) to identify knowledge and preferences of ACP, as well as preferences towards truth telling, healthcare autonomy, and end-of-life care of older adults living in the city of Beijing; (2) to explore the factors associated with those preferences.

## 2. Material and Methods

It was a cross-sectional survey conducted from August 6 to September 3, 2014. The study was performed at two communities in Chaoyang District, Beijing. The interviewers had training and practice sessions prior to the interviews. The face-to-face, semistructured interviews were conducted in the existing seniors clubs in the two communities. The seniors clubs were chosen because they provided the researchers with access to a large number of older persons in a short time frame. A total of 1098 people were in attendance in the seniors clubs during the period in which we visited; however, some people did not take the survey. A total of 921 participants completed the survey with a response rate of 83.9%. Of them, 21 participants were excluded from the analysis due to incompletion of the survey. A total of 900 surveys were included in the final analysis. Participants enrolled in the study were aged above 65 years, were able to understand the content of the questionnaire, and could communicate well with the interviewers. They were also willing to participate in the study. Older adults documented to have delirium, dementia, aphasia, and drowsiness were excluded from the study.

The questionnaire used in the study was reviewed and approved by the hospital directors and the hospital's ethical review board. It is composed of collection of participants' sociodemographic characteristics and five questions designed to delineate participants' awareness, preferences, and attitudes towards advance care planning, truth telling, healthcare autonomy, and end-of-life care. Sociodemographic characteristics including age, gender, religion, education level, marital status, living situation, monthly income, and social support conditions were collected. Social support conditions were reflected by whether older adults can get life support from their children and the number of close friends older adults have, from whom they can get support when they have difficulties.

Before asking the questions, participants were informed about the concept of ACP: “Advance care planning is defined as a communication and decision-making process that allows individuals to clarify their values and preferences for future care, and enables them to communicate their wishes to loved ones, surrogate decision makers and healthcare providers” [11]. After this introduction, they were asked the following questions: “Do you want to hear the real news regarding your own condition from the physician (Q1)?”; “Would you prefer to make your own healthcare decisions (Q2)?”; “Have you ever heard of an advanced care planning before (Q3)?”; “Do you want to document in an ACP so that your own values and preferences will be respected in case you become seriously ill (Q4)?”; “Are you willing to endure specific life-prolonging interventions (such as chronic ventilator and feeding tube) to avoid death in terms of irreversible conditions (Q5)?” Questions 1 and 2 were designed to reflect participants' attitudes towards healthcare autonomy, while questions 3 and 4 were designed to reflect participants' awareness and preferences towards ACP. Question 5 was designed to reflect participants' attitudes towards end-of-life care. These questions required a response of yes/no. If a participant did not want to answer certain questions, he/she could choose the option of “refuse to answer,” which was also recorded.

## 3. Statistical Analysis

Results were presented as means ± standard deviations for continuous variables. Categorical variables were reported as frequencies and percentages. Participants were classified into two groups: one group was those answering “yes” for each of the above-mentioned 5 questions, while the other group was those answering “no” for the questions. A Chi-square test was used to measure differences between the participants' sociodemographic characteristics in the two groups. Groups were tested for comparability on age, gender, religion, education level, marital status, living situation, income, and social support conditions. Values of *P* < 0.05 were considered statistically significant. All the analysis was performed using SPSS software version 16.0 for Windows (SPSS Inc., Chicago, IL, USA).

## 4. Results

Table 1 shows the sociodemographic characteristics of the participants. The mean age of participants was 74.99 ± 6.53 years; 53% (*n* = 477) of them were female. 79.8% (*n* = 718) of them were married, while 20.2% were single/divorced/widowed. 11.2% (*n* = 101) of the respondents lived alone. 70.4% (*n* = 634) of them listed being high school graduate or less for their education levels. Most of them had children (97.3%) and could get life support from their children (71.3%). The vast majority of them did not have a religion (97.6%).

The respondents' options for the five questions mentioned above were as follows—Table 2. Of the respondents, 80.9% wanted to hear the real news regarding their own condition from the physician, while 52.4% preferred to make their own healthcare decisions. Only a very small number of them (8.9%) preferred to endure life-prolonging interventions when faced with irreversible conditions. When introduced to the concept of ACP, the majority (78.3%) of them had not heard of it, and only 39.4% wanted to document in an ACP, whereas 41.9% did not. 18.7% of the respondents refused to answer this question.

According to results of the Chi-square test, Tables 3 [Table tab4] [Table tab5] [Table tab6]–7, survey respondents with higher education level (some college or more) had significantly higher proportion of having ever heard of an ACP, as well as preferring to document in an ACP, compared to those with education level of being high school graduate or less (23.6% versus 13.3%, *P* < 0.01; 60.3% versus 43.5%, *P* < 0.01, resp.). Those aged <70 years had higher proportion of having ever heard of an ACP, as well as refusing life-prolonging interventions to avoid death when faced with irreversible conditions, compared to those aged ≥70 years (21.9% versus 14.0%, *P* < 0.05; 91.0% versus 84.2%, *P* < 0.05). There was no difference in preferences for the questions between men and women. No statistical differences were found for gender, marital status, religion, income, having children, and support from children between those who answered “yes” and “no” for the above-mentioned questions.

## 5. Discussion

According to our knowledge, this study was the first attempt to investigate community-dwelling older persons' preferences and attitudes towards ACP, truth telling, healthcare autonomy, and end-of-life care, as well as explore the factors associated with those attitudes in mainland China. Consequently, such data will provide insight into our understanding of advance care planning and healthcare preferences of elderly community-dwellers in mainland China and serve as a foundation for later investigations on suitable ways of implementing ACP.

A low level of ACP awareness and preferences was apparent; very few participants had ever heard of an ACP, which can help to achieve their autonomy. However, the favoring of truth telling and self-determination by our participants was relatively high. Most of the participants preferred to hear the real news regarding their own condition from physician, and more than half of them wanted to make their own healthcare decisions. Only a very small portion of the participants expressed that they would endure life-prolonging interventions in terms of irreversible conditions. Previous literature showed that traditional Chinese societies were strongly family centered [12, 13]; healthcare decisions were often made by the family as a group, rather than by the individual, and the principle of autonomy played a lesser role in Chinese societies. In our study, encouragingly there was a significant proportion of elderly participants who favored healthcare autonomy and had negative attitudes towards life-prolonging interventions in terms of end-of-life care. When introduced to the concept of ACP, less than forty percent of respondents expressed that they would prefer to document in it. One possible explanation is that the concept is very new to them or unfamiliar. In mainland China, end-of-life care and the concept of ACP are not widely known or taught in the medical profession, and there are even fewer public promotion activities on these topics. Over one-third of participants refused to answer the question of “Are you willing to endure specific life-prolonging interventions to avoid death in terms of irreversible conditions (Q5)?” A possible explanation may be that these participants were hesitant to talk about death and end-of-life related decisions or they considered the topic taboo or were uncomfortable discussing it.

Factors influencing older people's attitudes towards ACP had been studied. We found that age and education level may be the influential factors. Survey respondents with higher education had significantly higher proportion of having heard of an ACP, as well as preferring to document in an ACP. Some previous studies also showed that higher education was associated with a greater awareness and preference for ACP [14, 15]. Chinese, Filipino, and Japanese studies have shown that a higher education level and degree of acculturation are associated with more positive views towards planning and communication regarding the end of life [16, 17]. From our perspective, participants with education level of being high school graduate or less may have reduced comprehension of ACP definitions and goals. Those aged <70 years had higher proportion of having heard of an ACP, as well as refusing life-prolonging interventions when faced with irreversible conditions in our study. A possible explanation is that these younger respondents may be less influenced by traditional Chinese culture compared to those much older respondents; therefore they were more likely to accept the views of ACP.

There have been very few studies regarding Chinese older adults' attitudes towards ACP, advance directives (AD), and palliative care. A cross-sectional study published in 2014 investigated advance directive and end-of-life care preferences among nursing home residents, which showed that most (95.3%) had never heard of AD, and only 31.5% preferred to make an AD. More than half (52.5%) would receive life-sustaining treatment if they sustained a life-threatening condition [18]. In a transnational survey, 62 patients from five hospitals of mainland China who are seriously ill were visited to study their attitudes towards end-of-life decisions. It showed that most respondents (80.3%) wanted to hear the truth directly if they were diagnosed with a terminal condition, but more than half of the respondents (55.7%) wanted continued treatment in irreversible conditions [19]. There have been even fewer studies focused on the attitudes of older Chinese people towards end-of-life decisions who migrated to and settled in Western countries. A Canadian study concluded that older Chinese people did not favor advance directives because they create negative thoughts by requiring people to contemplate their own demise [20]. Another study performed in the United States, however, showed that it is feasible to conduct a nurse-led educational seminar on ACP in a community-dwelling population of Chinese Americans; Chinese in this study were open to the topic and showed a willingness to learn about ACP [21].

A variety of factors that prevent elderly people from making advance care planning were identified in the literature, including irrelevance, reluctance to think about dying, lack of knowledge, feeling that planning is unnecessary because family knows what to do, and feeling that loved ones are unable or unwilling to discuss ACP [22, 23]. In particular, there are some obstacles towards implementing ACP in mainland China for the following reasons. The first reason is associated with culture, which shapes the way people deal with illness, suffering, and death, as well as the communications and decisions related to ACP [24]. In traditional Chinese culture, Confucianism and the relative importance placed on an individual's relationship with their family and society have a deep influence on decision-making, especially at the end of life [25, 26]. Family cohesion is highly valued and overrides the preferences and autonomy of an individual [27]. Moreover, the traditional Chinese superstition believed that death was a very sensitive issue, and mentioning it was sacrilegious and to be avoided [28]. Secondly, medical decision-making has been seen primarily as a duty of the family in order to protect the patient from the burden of making difficult choices about medical care [24]. It turned out to be that older adults are often excluded from decision-making.

Despite the obstacles mentioned above, the endeavor to promote ACP and palliative care has been made in mainland China in recent years. The foundation of Beijing Living Will Promotion Association, which was approved by the Beijing Civil Affairs Bureau in June 2013, was a landmark event in this regard [29]. Since its foundation, the organization had launched a website, called “Choice and Dignity,” to provide online advice on making living wills [30]. However, we should be aware that although there is a lot of evidence regarding ACP and end-of-life care discussions in Western countries, cultural attitudes towards such issues are different in Chinese societies [31]. Currently, there has been insufficient research to demonstrate the benefits of ACP to eastern Asian patients. For many Chinese older adults with the preference for making decisions as families and a relative unfamiliarity with ACP, the use of ACP may be discouraged. Moreover, patients' treatment preferences and values may change when their health changes, at the end of life and even during periods of stable health [32]. Therefore, it may be difficult and impractical to import ACP system and apply it directly in China. To implement ACP in mainland China, health professionals should conduct culturally specific advance care planning that is tailored to Chinese people's specific cultural attitudes and ethnic beliefs. Encouragingly, elderly people's favoring for self-determination as well as their negative attitudes towards life-sustaining treatment in terms of irreversible conditions in our study suggests that it is possible to initiate the topic concerning end-of-life care and ACP related issues to Chinese elderly citizens. Providing culture-sensitive knowledge, education, and communication regarding ACP is a feasible first step to promoting this health behavior in mainland China.

It is important to point out the limitations of this study. First, the participants in this study were small convenience samples. Thus, the generalization of the findings requires caution. Second, associated conditions of the older adults such as multimorbidity, functional status, and prior exposure to illness were not incorporated into the study, which may also be important determinants in ACP and its perceived relevance to individuals. Finally, the questionnaire, especially the five questions in the questionnaire to delineate participants' awareness, preferences, and attitudes towards advance care planning, truth telling, healthcare autonomy, and end-of-life care in this study, was designed by us and there is no previous literature to demonstrate its validity. Moreover, we used some hypothetical clinical scenarios in our questionnaire, for which reason responses to the survey might not accurately reflect what individuals would choose in reality and validity of the questionnaire needs to be further assessed.

## 6. Conclusion

This study identified the preferences and attitudes towards ACP and healthcare autonomy of community-dwelling older adults living in Beijing. Although the majority of elderly community-dwellers in this survey appeared to have medical autonomy and preferred comfort measures in terms of irreversible conditions, a low level of “Planning ahead” awareness and preferences was apparent. Given that the concept of advance care planning and knowledge of palliative care are not well understood in China, more effort is needed to step up public education in this regard. Moreover, to implement advance care planning in mainland China, it should be tailored to individuals' needs and feasible in the Chinese healthcare system and culture.

## Figures and Tables

**Table 1:** Sociodemographic characteristics of the participants (*n* = 900).

Variables	*N*	Percent (%)
Gender		
Male	423	47
Female	477	53
Age	74.99 ± 6.53	
<70 years	267	29.7
≥70 years	633	70.3
Education		
High school graduate or less	634	70.4
Some college or more	266	29.6
Living situation		
Lives alone	104	11.5
Lives with someone	796	88.5
Marital status		
Married	718	79.8
Single/widowed/divorced	182	20.2
Religiosity		
No	878	97.6
Yes	22	2.4
Monthly income (RMB)		
<1300	36	4.0
1300–5000	766	85.1
>5000	78	8.6
Unknown	20	2.2
Have children		
No	24	2.7
Yes	876	97.3
Support from children		
Yes	642	71.3
No	208	23.1
Refuse to answer	50	5.6
Number of close friends to provide life support and help		
0	235	26.1
1–5	444	49.3
≥6	174	19.3
Unknown	47	5.3

**Table 2:** Participants' answers to the five questions regarding truth telling, ACP, healthcare autonomy, and end-of-life care.

Number	Sample questions	Yes *N* (%)	No *N* (%)	Refuse to answer *N* (%)	Total *N* (%)
1	“Do you want to hear the real news regarding your own condition from the physician?”	728 (80.9)	92 (10.2)	80 (8.9)	900 (100)

2	“Would you prefer to make your own healthcare decisions?”	472 (52.4)	312 (34.7)	116 (12.9)	900 (100)

3	“Have you ever heard of an ACP?”	138 (15.3)	705 (78.3)	57 (6.4)	900 (100)

4	“Do you want to document in an ACP so that your own values and preferences will be respected in case you become seriously ill?”	355 (39.4)	377 (41.9)	168 (18.7)	900 (100)

5	“Are you willing to endure specific life-prolonging interventions (such as chronic ventilator and feeding tube) to avoid death when faced with irreversible conditions?”	79 (8.9)	503 (55.8)	318 (35.3)	900 (100)

**Table 3:** Comparison of sociodemographic characteristics of respondents answering “yes” and “no” to question 1 (*N* = 820).

Variables	All (*N* = 820)	Yes (*n* = 728)	No (*n* = 92)	*χ* ^2^ (*P* value)
Gender				
Male	386	347	39	0.912 (0.34)
Female	434	381	53	
Age				
<70 years	245	215	30	0.369 (0.544)
≥70 years	575	513	62	
Education				
High school graduate or less	581	507	74	4.606 (0.032)
Some college or more	239	221	18	
Living condition				
Lives alone	92	84	8	0.288 (0.591)
Lives with someone	728	644	84	
Marital status				
Married	655	585	70	1.308 (0.253)
Unmarried/widowed/divorced	165	143	22	
Religiosity				
No	797	706	91	1.094 (0.499)
Yes	23	22	1	
Monthly income (RMB)				
<1300	29	23	6	4.031 (0.231)
1300–5000	703	630	73	
>5000	70	60	10	
Unknown	18	15	3	
Have children				
No	23	23	0	(2.990) 0.097
Yes	797	705	92	
Support from children				
Yes	578	516	62	
No	198	185	13	
Refuse to answer	44	27	17	
Number of close friends to provide life support and help				
0	208	179	29	3.045 (0.385)
1–5	413	371	42	
≥6	160	141	19	
Unknown	39	37	2	

**Table 4 tab4:** Comparison of sociodemographic characteristics of respondents answering “yes” and “no” to question 2 (*N* = 784).

Variables	Total sample (*N* = 784)	Yes (*n* = 472)	No (*n* = 312)	*χ* ^2^ (*P* value)
Gender				
Male	371	225	146	0.058 (0.81)
Female	413	247	166	
Age				
<70 years	241	153	88	1.564 (0.211)
≥70 years	543	319	224	
Education				
High school graduate or less	554	326	228	1.347 (0.262)
Some college or more	230	146	84	
Living condition				
Lives alone	85	54	31	0.229 (0.632)
Lives with someone	699	418	281	
Marriage				
Married	628	381	247	0.247 (0.619)
Unmarried/widowed/divorced	156	91	65	
Religiosity				
No	764	456	308	3.317 (0.103)
Yes	20	16	4	
Monthly income (RMB)				
<1300	28	15	13	3.325 (0.358)
1300–5000	673	409	264	
>5000	66	41	25	
Unknown	17	7	10	
Have children				
No	22	11	11	0.984 (0.321)
Yes	762	461	301	
Support from children				
Yes	546	343	203	2.590 (1.108)
No	195	109	86	
Refuse to answer	43	20	23	
Number of close friends to provide life support and help				
0	199	115	84	4.899 (0.179)
1–5	399	232	167	
≥6	152	104	48	
Unknown	34	21	13	

**Table 5 tab5:** Comparison of sociodemographic characteristics of respondents answering “yes” and “no” to question 3 (*N* = 843).

Variables	Total sample (*N* = 843)	Yes (*n* = 138)	No (*n* = 705)	*χ* ^2^ (*P* value)
Gender				
Male	395	67	328	0.19 (0.663)
Female	448	71	377	
Age				
<70 years	256	56	200	8.138 (0.04)
≥70 years	587	82	505	
Education				
High school graduate or less	593	79	514	13.569 (0.000)
Some college or more	250	59	191	
Living condition				
Lives alone	94	17	77	0.261 (0.609)
Lives with someone	749	121	628	
Marriage				
Married	676	112	564	0.071 (0.789)
Unmarried/widowed/divorced	167	26	141	
Religiosity				
No	821	136	685	0.466 (0.495)
Yes	22	2	20	
Monthly income (RMB)				
<1300	30	1	29	6.236 (0.101)
1300–5000	721	117	604	
>5000	73	17	56	
Unknown	19	3	16	
Have children				
No	23	6	17	1.631 (0.202)
Yes	820	132	688	
Support from children				
Yes	592	94	498	1.792 (0.408)
No	201	32	169	
Refuse to answer	50	12	38	
Number of close friends to provide life support and help				
0	212	28	184	4.102 (0.251)
1–5	425	71	354	
≥6	165	34	131	
Unknown	41	5	36	

**Table 6 tab6:** Comparison of sociodemographic characteristics of respondents answering “yes” and “no” to question 4 (*N* = 732).

Variables	Total sample (*N* = 732)	Yes (*n* = 355)	No (*n* = 377)	*χ* ^2^ (*P* value)
Gender				
Male	350	177	173	0.155 (0.282)
Female	382	178	204	
Age				
<70 years	226	124	102	5.311 (0.021)
≥70 years	506	231	275	
Education				
High school graduate or less	513	223	290	17.352 (0.000)
Some college or more	219	132	87	
Living condition				
Lives alone	81	45	36	1.356 (0.244)
Lives with someone	651	310	341	
Marriage				
Married	583	289	294	1.322 (0.250)
Unmarried/widowed/divorced	149	66	83	
Religiosity				
No	710	344	366	0.303 (0.582)
Yes	22	11	11	
Monthly income (RMB)				
<1300	25	9	16	3.486 (0.323)
1300–5000	627	302	325	
>5000	65	37	28	
Unknown	15	7	8	
Have children				
No	19	11	8	0.671 (0.413)
Yes	713	344	369	
Support from children				
Yes	514	257	257	2.296 (0.317)
No	175	79	96	
Refuse to answer	43	19	24	
Number of close friends to provide life support and help				
0	184	88	96	9.847 (0.020)
1–5	367	164	203	
≥6	149	81	68	
Unknown	32	22	10	

**Table 7:** Comparison of sociodemographic characteristics of respondents answering “yes” and “no” to question 5 (*N* = 582).

Variables	Total sample (*N* = 582)	Yes (*n* = 79)	No (*n* = 503)	*χ* ^2^ (*P* value)
Gender				
Male	276	37	239	0.013 (0.91)
Female	306	42	264	
Age				
<70 years	189	17	172	5.503 (0.025)
≥70 years	393	62	331	
Education				
High school graduate or less	397	58	339	1.142 (0.285)
Some college or more	185	21	164	
Living condition				
Lives alone	62	6	56	0.855 (0.355)
Lives with someone	520	73	447	
Marriage				
Married	468	68	400	1.861 (0.172)
Unmarried/widowed/divorced	114	11	103	
Religiosity				
No	562	76	486	0 (1.000)
Yes	20	3	17	
Monthly income (RMB)				
<1300	16	2	14	1.704 (0.636)
1300–5000	508	71	437	
>5000	48	6	42	
Unknown	10	0	10	
Have children				
No	17	3	14	0.026 (0.873)
Yes	565	76	489	
Support from children				
Yes	406	61	345	0.210 (0.350)
No	150	16	134	
Refuse to answer	26	2	24	
Number of close friends to provide life support and help				
0	137	22	115	17.117 (0.001)
1–5	293	43	250	
≥6	127	12	115	
Unknown	25	2	23	
